# Fluoridation cessation and oral health equity: a 7-year post-cessation study of Grade 2 schoolchildren in Alberta, Canada

**DOI:** 10.17269/s41997-022-00654-4

**Published:** 2022-07-07

**Authors:** Lindsay McLaren, Steven K. Patterson, Peter Faris, Guanmin Chen, Salima Thawer, Rafael Figueiredo, Cynthia Weijs, Deborah A. McNeil, Arianna Waye, Melissa L. Potestio

**Affiliations:** 1 Department of Community Health Sciences, Cumming School of Medicine, University of Calgary, 3280 Hospital Dr. NW, Calgary, AB Canada; 2 School of Dentistry, Faculty of Medicine and Dentistry, University of Alberta, Edmonton, AB Canada; 3 Data & Analytics, Alberta Health Services, Calgary, AB Canada; 4 Faculty of Education, Western University, London, ON Canada; 5 Provincial Oral Health Office, Provincial Population and Public Health, Alberta Health Services, Calgary, AB Canada; 6 Department of Geography, Faculty of Arts, University of Calgary, Calgary, AB Canada; 7 Strategic Clinical Networks, Alberta Health Services, Calgary, AB Canada; 8 Health Innovation & Excellence, Provincial Clinical Excellence, Alberta Health Services, Calgary, AB Canada

**Keywords:** Fluoridation, Health equity, Dental caries, Public health, Enrichissement en fluor, équité en santé, caries dentaires, santé publique

## Abstract

**Objective:**

Community water fluoridation, because of its universal scope and passive mechanism of uptake, is one component of a multifaceted approach to promoting equity in dental health. The objective of this study was to examine social inequities in children’s dental health in the Canadian cities of Calgary (fluoridation cessation in 2011) and Edmonton (still fluoridated).

**Methods:**

We analyzed data from surveys of population-based samples of Grade 2 (approx. age 7) children in Calgary in 2009/2010 (pre-cessation; *n*=557) and in both Calgary and Edmonton in 2013/2014 (Calgary, *n*=3230; Edmonton, *n*=2304) and 2018/2019 (Calgary, *n*=2649; Edmonton, *n*=2600) (post-cessation). We estimated associations between several socioeconomic indicators and dental caries indicators (i.e., dental caries experience [*deft*, *DMFT*] and untreated decay in two or more teeth [*untreated decay*]) using zero-inflated Poisson, binary logistic regression, and the concentration index of inequality. We compared those associations over time (between survey waves) and between cities at post-cessation.

**Results:**

Persistent social inequities in *deft* and *untreated decay* were evident; for example, having no dental insurance was significantly associated with higher odds of *untreated decay* across city and survey wave. In most (but not all) cases, differences between cities and survey waves were consistent with an adverse effect of fluoridation cessation on dental health inequities. For example, the association between no dental insurance and higher odds of *untreated decay* in Calgary was greater in 2018/2019 (later post-cessation) than in 2009/2010 (pre-cessation; odds ratio [OR] for comparison of coefficients = 1.89 [1.36–2.63], *p*<0.001) and 2013/2014 (early post-cessation; OR for comparison of coefficients = 1.67 [1.22–2.28], *p*=0.001); that same association in 2018/2019 was greater in Calgary (fluoridation cessation) than in Edmonton (still fluoridated) (OR for comparison of coefficients = 1.44 [1.03–2.02], *p*=0.033).

**Conclusion:**

Social inequities in dental caries were present in both Calgary and Edmonton. Those inequities tended to be worse in Calgary where fluoridation was ceased. Our findings may be relevant to other settings where income inequality is high, dental services are costly, and dental public health infrastructure is limited.

## Résumé

**Objectif:**

En raison de sa portée universelle et de son mécanisme de réception passif, la fluoration de l’eau des communautés s’inscrit dans une démarche multidimensionnelle de promotion de l’équité en santé dentaire. Notre étude visait à examiner les iniquités sociales en santé dentaire chez les enfants dans les villes canadiennes de Calgary (où la fluoration a cessé en 2011) et d’Edmonton (où l’eau est encore enrichie en fluor).

**Méthode:**

Nous avons analysé les données d’enquêtes menées auprès d’échantillons populationnels d’élèves de 2^e^ année (environ 7 ans) à Calgary en 2009-2010 (avant l’arrêt; *n* = 557), et à Calgary et Edmonton en 2013-2014 (Calgary, *n* = 3 230; Edmonton, *n* = 2 304) et en 2018-2019 (Calgary, *n* = 2 649; Edmonton, *n* = 2 600) (après l’arrêt). Nous avons estimé les associations entre plusieurs indicateurs socioéconomiques et indicateurs de caries dentaires (c.-à-d. l’expérience de caries dentaires [*dceo*, *DCMO*] et de dégradation non traitée dans deux dents ou plus [*dégradation non traitée*]) à l’aide de la régression de Poisson à surreprésentation de zéros, de la régression logistique binaire et de l’indice de concentration des inégalités. Nous avons comparé ces associations dans le temps (entre les cycles de l’enquête) et entre les deux villes après l’arrêt de la fluoration.

**Résultats:**

Des iniquités sociales persistantes selon l’indice *dceo* [dents cariées, extraites et obturées] et la *dégradation non traitée* étaient manifestes; par exemple, l’absence d’assurance dentaire présentait une corrélation significative avec une probabilité accrue de *dégradation non traitée* d’une ville à l’autre et d’un cycle à l’autre de l’enquête. Dans la plupart des cas (mais pas tous), les différences entre les villes et entre les cycles de l’enquête correspondaient à un effet indésirable de l’arrêt de la fluoration sur les iniquités en santé dentaire. Par exemple, l’association entre l’absence d’assurance dentaire et la probabilité accrue de *dégradation non traitée* à Calgary était plus importante en 2018-2019 (longtemps après l’arrêt) qu’en 2009-2010 (avant l’arrêt; rapport de cotes [RC] pour comparaison des coefficients = 1,89 [1,36-2,63], *p* < 0,001) et qu’en 2013-2014 (peu après l’arrêt; RC pour comparaison des coefficients = 1,67 [1,22-2,28], *p* = 0,001); cette même association en 2018-2019 était plus importante à Calgary (où la fluoration a cessé) qu’à Edmonton (où l’eau est encore enrichie en fluor) (RC pour comparaison des coefficients = 1,44 [1,03-2,02], *p* = 0,033).

**Conclusion:**

Des iniquités sociales relativement aux caries dentaires étaient présentes tant à Calgary qu’à Edmonton. Ces iniquités avaient tendance à être plus importantes à Calgary, où la fluoration a cessé. Nos constatations pourraient être pertinentes dans les autres endroits où l’inégalité des revenus est élevée, où les soins dentaires coûtent cher et où les infrastructures en santé publique dentaire sont limitées.

## Introduction

Community water fluoridation (“fluoridation”) is the controlled adjustment of the fluoride content in public drinking water supplies to a level recommended for preventing tooth decay (Burt & Eklund, 1999). Because of its universal scope and passive mechanism of uptake, fluoridation has the potential to be equitable in its impact—that is, to benefit everyone but especially those with poorer dental health and/or less access to other avenues of prevention and health promotion (McLaren et al., 2010).

There are significant social inequities in dental health (Schwendicke et al., 2015). Social inequities in health may be defined as unfair and avoidable differences in health outcomes between social groups that are driven by the inequitable distribution of power, money, and resources and favour socioeconomically advantaged groups (Commission on Social Determinants of Health, 2008). Consistent with international literature (Schwendicke et al., 2015), we have observed inequities in dental caries (tooth decay), by socioeconomic circumstances and by ethnicity, in our setting of Alberta, Canada (Shi et al., 2018; 2021).

Promoting dental health equity requires a multifaceted approach which recognizes that health outcomes are distributed along a social gradient in the population (Graham, 2004). Fluoridation, to which approximately 39% of Canadians are presently exposed (Public Health Agency of Canada, 2017), constitutes one element of such an approach. Indeed, an equitable effect of fluoridation on dental caries has been borne out in cross-sectional studies in (for example) Canada (McLaren & Emery, 2012), Britain (Jones & Worthington, 2000), Australia (Slade et al., 1996), New Zealand (Treasure & Dever, 1994), South Korea (Cho et al., 2014), and the United States (Sanders et al., 2019). The present study builds on the existing literature by examining social inequities in dental caries in the context of fluoridation cessation, which occurred in Calgary, Canada, in 2011. Research on social inequities in dental caries in the context of fluoridation cessation is rare (McLaren & Singhal, 2016; Meyer et al., 2018).

The objective of the present study was to examine social inequities in dental caries (i.e., associations between socioeconomic indicators and dental caries indicators) among schoolchildren in Calgary (fluoridation cessation in 2011) and Edmonton (still fluoridated), including to compare those associations between survey waves over time and between cities. In a previous study (McLaren et al., 2016a), we found that inequities in dental caries in Calgary (fluoridation cessation), by dental insurance status and by small area material deprivation, were more apparent at early post-cessation (2013/2014) than at pre-cessation (2009/2010). The present study builds on that earlier research by (1) extending (to 2018/2019) the time frame of the previous evaluation in Calgary to consider whether our earlier findings persist, (2) including post-cessation data available from Edmonton, a fluoridated comparison city, and (3) considering a broader range of socioeconomic indicators available at post-cessation, namely, household education and dwelling tenure, in addition to dental insurance status (for which a more detailed version was available at post-cessation) and small area material deprivation (these are defined below). Our study sheds light on the practical question of whether or the extent to which other interventions (e.g., other dental public health programs) in our setting have been adequate to offset the lack of fluoridation in Calgary. This is informative for other communities that have ceased, or are revisiting, fluoridation (McLaren & Singhal, 2016).

Elsewhere (McLaren et al., 2021), we considered the average or overall effects of fluoridation cessation on dental health of Grade 2 schoolchildren in Calgary and Edmonton, Canada. The present study, which uses data from the same project, complements that paper by focusing specifically on dental health equity.

## Methods

### Design, study sample, and data collection

**Fig. 1:**
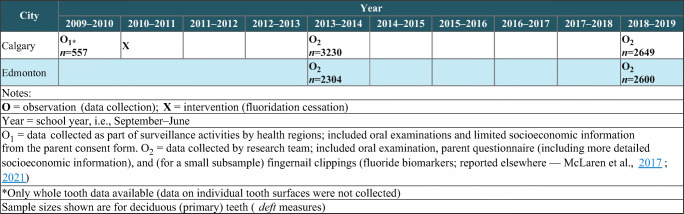
Schematic of study design

The study design is depicted in Figure 1. We analyzed data from population-based samples of schoolchildren gathered in 2009/2010 (pre-cessation) in Calgary only, and in 2013/2014 (early post-cessation) and 2018/2019 (later post-cessation) in both Calgary and Edmonton, Canada. As described in more detail elsewhere (McLaren et al., 2021), Calgary and Edmonton are the two largest cities in the province of Alberta, Canada (2016 population approximately 1.24 million and 932,500, respectively), and census data confirm that they are reasonably similar in important sociodemographic respects, with some indication of lower socioeconomic circumstances in Edmonton (for example, the prevalence of low-income status was 12.9% in Calgary and 16.1% in Edmonton according to the 2016 census) (Statistics Canada, 2019). Fluoridation was ceased in Calgary in 2011 after having been in place since 1991. In Edmonton, fluoridation began in 1967 and remains in place (McLaren et al., 2016a; 2017; 2016b; 2021).

Details of the study setting, sampling, and data collection are described elsewhere (McLaren et al., 2016a; 2021). Briefly, the target population was Grade 2 schoolchildren (approx. age 7) enrolled in public or separate school systems in the two cities. These two school systems in 2018/2019 included over 90% of all Alberta schoolchildren. A population-based sample was drawn from each city using a stratified random sampling procedure where strata were defined based on the median household income of the dissemination area in which the school was located. Within sampled schools, all children in Grade 2 were invited to participate. Primary sampling unit and sampling weights were developed for each survey and applied to all analyses; these account for clustered sampling. The sampling weights account for the probability of selection and the probability of non-response. This approach allowed us to handle missing observations within the framework of our survey sampling approach rather than, for example, having to estimate differences between our samples and the target populations (Little & Rubin, 1987). Response rates in 2018/2019 for schools, and for students in participating schools (those with both oral examination and questionnaire data), were 53.8% and 44.5% (Edmonton) and 46.7% and 43.7% (Calgary). Response rates in 2013/2014 for schools, and for students in participating schools (those with both oral examination and questionnaire data), were 54.1% and 47.0% (Edmonton) and 57.3% and 49.1% (Calgary). The overall response rate in 2009/2010 (Calgary only) was 81%. The 2009/2010 data were collected by health regions as part of surveillance activities, whereas the 2013/2014 and 2018/2019 data were collected as part of a research project; otherwise, the surveys were designed to be as comparable as possible.

Dental caries data were collected via an oral examination conducted at school by trained and calibrated assessment teams. In 2009/2010 (Calgary only), limited socioeconomic information was collected as part of the parent consent form. In 2013/2014 (Calgary and Edmonton) and 2018/2019 (Calgary and Edmonton), socioeconomic information was collected via a questionnaire voluntarily completed by parents. In 2009/2010 (Calgary only), of *n*=559 with oral exam data, *n*=2 were excluded due to missing data on school code or weighted ID, leaving an analytic sample of *n*=557. In 2013/2014, of *n*=3257 (Calgary) and *n*=2328 (Edmonton) with both dental exam and questionnaire data, *n*=27 (Calgary) and *n*=24 (Edmonton) were excluded due to missing data on school code or weighted ID, leaving an analytic sample of *n*=3230 (Calgary) and *n*=2304 (Edmonton). In 2018/2019, of *n*=2652 (Calgary) and *n*=2614 (Edmonton) with both dental exam and questionnaire data, *n*=3 (Calgary) and *n*=14 (Edmonton) were excluded due to missing data on school code or weighted ID, leaving an analytic sample of *n*=2649 (Calgary) and *n*=2600 (Edmonton).^1^

The study received approval from the Conjoint Health Research Ethics Board at the University of Calgary and the Health Research Ethics Board at the University of Alberta (information for 2018/2019 iteration: REB18-0273 and Pro00081226 respectively).

### Study variables

Outcome variables were (1) *deft* and (2) *DMFT*, which are standard indices of dental caries (tooth decay) experience in primary and permanent teeth respectively and (3) *untreated decay*, which refers to the presence of two or more decayed teeth, primary or permanent.^2^ To create these indices, each tooth is categorized as having no decay experience, having decay (d, D), being extracted or missing due to decay (e, M), or having fillings (f, F) (World Health Organization, 2013). Both *deft* and *DMFT* include treated (fillings, extractions) and untreated decay, which could show different associations with socioeconomic indicators depending on access to treatment services; for that reason, we also examined *untreated decay* (presence or absence of two or more decayed teeth, based on d and D) separately. The *deft* and *DMFT* are count variables with large numbers of zero values (children with no decay experience); we therefore modelled them both as count variables, and as presence (1 or more) versus absence (0).

Different socioeconomic indicators were available at different times (see below); altogether these included dental insurance status; small area material deprivation (Pampalon et al., 2009); highest household educational attainment (i.e., high school graduation or post-secondary diploma/certificate or less, completed bachelor’s degree, completed degree or certificate above bachelor’s or higher); and dwelling tenure (i.e., rented or other, owned with mortgage, owned with no mortgage). Household educational attainment and dwelling tenure were only available at post-cessation (2013/2014 and 2018/2019). The consideration of multiple socioeconomic indicators permits some insight into consistency and specificity of effects.

For dental insurance, a two-category variable—i.e., presence (any type) versus absence—was available for Calgary in 2009/2010 (pre-cessation). For Calgary and Edmonton in 2013/2014 and 2018/2019 (post-cessation), we had a three-category variable: no insurance; public insurance (i.e., provincial or federal government program); private/employer insurance (i.e., privately purchased or employer-sponsored) (Alberta Health Services, 2018).^3^

The small area material deprivation index is a composite variable based on age- and sex-adjusted data from the national census, namely, average individual income, employment to population ratio, and proportion without a high school diploma or equivalent. It describes dissemination areas, which are small geographic units (400–700 population) used in the census (Statistics Canada, n.d.). We used the version of the index based on the 2016 census (the Canadian census is administered quinquennially), which we applied to all waves of data (i.e., 2009/2010, 2013/2014, and 2018/2019) for consistency. Each participant was assigned an index value by linking their home postal code to the corresponding dissemination area. Material deprivation was expressed as a continuous variable based on factor analysis by those who created the index (Institut national de santé publique du Québec, 2019) and as a three-category variable where tertiles were created based on the full Alberta population and then applied to our samples.

### Analysis

Associations were tested separately for each socioeconomic indicator. We first examined associations in Calgary (2009/2010, 2013/2014, and 2018/2019) and Edmonton (2013/2014, 2018/2019) between dental caries indicators and the two socioeconomic indicators that were available in the pre-cessation Calgary survey (i.e., dental insurance [presence vs absence] and small area material deprivation [tertiles, with low deprivation as the reference group]). We used zero-inflated Poisson (for *deft* and *DMFT* as count variables) or binary logistic (for presence/absence of *deft* and *DMFT* and for *untreated decay*) regression.^4^ We then compared the associations within each city over time (i.e., 2018/2019 compared to 2013/2014 and 2009/2010 survey waves in Calgary, and 2018/2019 compared to 2013/2014 survey waves in Edmonton), and—for post-cessation only—between cities at each time point (i.e., 2018/2019 and 2013/2014 survey waves) using the Wald test (Harrell Jr., 2016).

Second, for each survey wave for Calgary and Edmonton, we estimated the concentration index of inequality for each dental caries indicator (i.e., *deft*, *DMFT*, *untreated decay*) by material deprivation (continuous variable). The concentration index complements the other measures of association (described above) by providing a different way of conceptualizing inequality; namely, it quantifies the extent to which a health problem is disproportionately concentrated in lower (or higher) socioeconomic groups (in our case, concentrated among those who live in areas of higher material deprivation).^5^ Whether the concentration indices in 2018/2019 differed from those estimated in previous waves (i.e., 2009/2010 for Calgary only; 2013/2014 for both Calgary and Edmonton) was tested using a statistical method developed by Clogg et al. (1995).

Third, associations between dental caries indicators and the socioeconomic indicators available at post-cessation only (i.e., dental insurance [3 categories], household education, and dwelling tenure) were examined using zero-inflated Poisson (for *deft* and *DMFT* as count variables) or binary logistic (for presence/absence of *deft* and *DMFT* and for *untreated decay*) regression in Calgary and Edmonton separately. As above, these associations were compared between waves (2013/2014 and 2018/2019) for each city, and between city (Calgary and Edmonton) for each post-cessation wave, using the Wald test (Harrell Jr., 2016).

To maximize the sample size available for each analysis, pairwise deletion was used. A sample size calculation was not conducted for the analyses presented here; it was, however, conducted for our main effects analysis which is reported elsewhere (McLaren et al., 2021).

## Results

Weighted descriptive statistics (i.e., point estimates by city and data point) for all study variables, on which the following model results are based, are shown in Table  1.

**Table 1:** Weighted descriptive statistics for all study variables

**Variable**	**Mean or %, 95% confidence interval,** ***n***					
	**Calgary** **(Fluoridation cessation in 2011)**			**Edmonton** **(Still fluoridated)**		
	**2009/2010**	**2013/2014**	**2018/2019**	**2009/2010**	**2013/2014**	**2018/2019**
Dental caries indicators (outcome variables)						
*deft* (mean)	2.2 (1.9–2.6), *n*=557	2.7 (2.5–2.8), *n*=3230	3.6 (3.4–3.8), *n*=2649	--	2.8 (2.62–3.0), *n*=2304	2.6 (2.4–2.8), *n*=2600
*deft* (% presence)	52.7 (47.5–57.9), *n*=557	56.6 (54.7–58.6), *n*=3230	64.8 (62.3–67.3), *n*=2649	--	58.7 (56.1–61.2), *n*=2304	55.1 (52.3–57.8), *n*=2600
*DMFT* (mean)	0.19 (0.11–0.28), *n*=551	0.12 (0.10–0.13), *n*=3182	0.33 (0.28–0.37), *n*=2627	--	0.17 (0.14–0.20), *n*=2260	0.21 (0.17–0.25), *n*=2569
*DMFT* (% presence)	10.3 (7.0–14.8), *n*=551	7.7 (6.8–8.8), *n*=3182	18.1 (16.1–20.3), *n*=2627	--	9.3 (7.9–11.0), *n*=2260	13.6 (11.5–16.0), *n*=2569
*Untreated decay* (%)	9.9 (7.5–12.9), *n*=551	13.6 (12.1–15.2), *n*=3182	18.8 (16.8–21.0), *n*=2627	--	21.0 (18.9–23.3), *n*=2260	21.5 (19.2–24.0), *n*=2569
Dental insurance*						
*No insurance* (%)	20.6 (16.9–24.8), *n*=98	16.8 (15.2–18.5), *n*=548	13.8 (12.3–15.4), *n*=350	--	18.9 (16.8–21.1), *n*=403	15.6 (14–17.4), *n*=380
*Public insurance* (%)	79.4 (75.2–83.1), *n*=430	9.6 (8.1–11.4), *n*=305	10.6 (8.8–12.7), *n*=226	--	11.7 (9.6–14.1), *n*=237	10.3 (8.5–12.5), *n*=227
*Private/employer insurance* (%)		73.6 (70.8–76.2), *n*=2311	75.7 (72.9–78.2), *n*=1993	--	69.5 (65.9–72.8), *n*=1556	74.0 (71.4–76.5), *n*=1907
Small area material deprivation**						
*High (%)*	30.9 (18.3–47.0), *n*=143	33.4 (28.2–39.0), *n*=1070	36.0 (30.1–42.2), *n*=743	--	49.7 (44.3–55.1), *n*=1024	45.5 (40.0–51.2), *n*=1031
*Middle (%)*	36.1 (23.2–51.4), *n*=202	30.7 (25.9–35.9), *n*=950	31.8 (26.9–37.1), *n*=884	--	31.5 (27.9–35.4), *n*=695	29.2 (25.4–33.3), *n*=739
*Low (%)*	33.0 (21.6–46.9), *n*=204	35.9 (30.5–41.7), *n*=1039	32.3 (27.2–37.8), *n*=937	--	18.8 (14.6–23.8), *n*=435	25.3 (20.2–31.2), *n*=689
Highest household educational attainment						
*Low* (%)	--	40.1 (36.4–43.9), *n*=1232	39.0 (35.3–42.8), *n*=907	--	51.8 (47.6–55.9), *n*=1091	47.0 (43.1–51), *n*=1121
*Middle* (%)	--	29.5 (27.4–31.7), *n*=905	31.6 (29.4–33.8), *n*=847	--	23.9 (21.6–26.3), *n*=522	26.9 (24.9–29.1), *n*=723
*High* (%)	--	30.4 (27.0–34.0), *n*=955	29.5 (26.3–32.9), *n*=808	--	24.4 (21.2–27.9), *n*=532	26.1 (22.9–29.5), *n*=685
Dwelling tenure						
*Rented or other* (%)	--	26.0 (23.4–28.9), *n*=824	28.6 (25.2–32.4), *n*=650	--	35.8 (31.7–40.2), *n*=718	32.6 (29.3–36.2), *n*=704
*Owned, with mortgage* (%)	--	63.6 (60.7–66.4), *n*=1941	64.1 (60.6–67.4), *n*=1680	--	55.2 (51.3–59.0), *n*=1220	60.5 (57.2–63.7), *n*=1582
*Owned, no mortgage* (%)	--	10.4 (8.9–12.0), *n*=325	7.3 (6.1–8.8), *n*=204	--	9.0 (7.6–10.6), *n*=197	6.9 (5.8–8.1), *n*=180

*deft* = number of decayed, extracted (due to decay), and filled primary teeth; *DMFT* = number of decayed, missing (due to decay), and filled permanent teeth; *untreated decay* = presence of two or more teeth (primary or permanent) with untreated decay

*For 2009/2010, and for the Calgary-specific analysis (i.e., 2009/2010, 2013/2014, and 2018/2019), a two-category version of dental insurance is available: presence versus absence of dental insurance, where presence refers to any type of insurance (i.e., private, employer-sponsored, or public)

Household education categories: low: ≤ high school grad or post-secondary diploma or certificate; middle: completed bachelor’s degree; high: completed degree or certificate higher than bachelor’s

**Categories for material deprivation are tertiles from the continuous deprivation variable, which was computed for the Alberta population and applied to our sample, hence they depart from equal size. High = most deprived (tertile 3); low = least deprived (tertile 1)

Table 2 shows results of regression analyses in Calgary (2009/2010, 2013/2014, and 2018/2019) and Edmonton (2013/2014 and 2018/2019) for the subset of socioeconomic variables that were available in Calgary at pre-cessation (2009/2010).

**Table 2:** Weighted estimates from regression analyses (zero-inflated Poisson or logistic) among Grade 2 schoolchildren in Calgary and Edmonton in 2009/2010 (Calgary only), 2013/2014 (Calgary and Edmonton), and 2018/2019 (Calgary and Edmonton) using the limited socioeconomic variables available in Calgary at pre-cessation (2009/2010). Dental caries measures (*deft*, *DMFT*, *untreated decay*) regressed on (1) dental insurance status (yes/no) and (2) small area material deprivation (tertiles)

**Outcome variable**	**Predictor variable**	**Estimate (RR or OR), 95% confidence interval,** ***n***					
		**Calgary** **(Fluoridation cessation in 2011)**			**Edmonton** **(Still fluoridated)**		
		**2009/2010**	**2013/2014**	**2018/2019**	**2009/2010**	**2013/2014**	**2018/2019**
*Dental insurance (reference: presence of insurance)*							
*deft* count (RR)	No dental insurance	1.05 (0.95–1.15), *n*=528	0.94 (0.86–1.03), *n*=3164^a^	1.05 (0.97–1.14), *n*=2569	--	1.16 (1.06–1.27)*^a^, *n*=2196	1.08 (0.97–1.20), *n*=2514
*deft* presence (OR)	No dental insurance	1.39 (1.03–1.87)*, *n*=528	1.13 (0.92–1.39), *n*=3164	1.28 (0.99–1.65), *n*=2569	--	1.35 (1.05–1.73)*, *n*=2196	1.31 (1.02–1.7)*, *n*=2514
*DMFT* count (RR)	No dental insurance	0.88 (0.68–1.15), *n*=522	1.56 (1.04–2.33)*, *n*=3120	0.89 (0.65–1.20)^b^, *n*=2548	--	1.36 (0.90–2.05), *n*=2153	1.40 (1.03–1.90)*^b^, *n*=2486
*DMFT* presence (OR)	No dental insurance	1.07 (0.59–1.92), *n*=522	1.16 (0.77–1.74), *n*=3120	1.10 (0.79–1.52), *n*=2548	--	1.44 (0.93–2.23), *n*=2153	1.3 (0.9–1.88), *n*=2486
*Untreated decay* (OR)	No dental insurance	1.76 (1.38–2.26)*^c^, *n*=522	2.0 (1.58–2.53)*^d^, *n*=3120	3.34 (2.64–4.22)*^c,d,e^, *n*=2548	--	2.06 (1.56–2.73)*, *n*=2153	2.31 (1.82–2.95)*^e^, *n*=2486
*Small area material deprivation (reference: low deprivation)*							
*deft* count (RR)	Middle deprivation	1.07 (0.88–1.3), total *n*=549	1.16 (1.06–1.27)*, total *n*=3059	1.06 (0.99–1.14), total *n*=2654	--	1.05 (0.93–1.18), total *n*=2154	1.14 (1.0–1.28)*, total *n*=2459
	High deprivation	1.2 (1.01–1.44)*	1.2 (1.09–1.31)*	1.13 (1.03–1.23)*	--	1.20 (1.09–1.32)*	1.23 (1.10–1.37)*
*deft* presence (OR)	Middle deprivation	1.43 (1.08–1.9)*, total *n*=549	1.2 (0.99–1.46), total *n*=3059	1.16 (0.96–1.41), total *n*=2654	--	1.16 (0.91–1.48), total *n*=2154	1.13 (0.88–1.44), total *n*=2459
	High deprivation	1.65 (1.01–2.69)*	1.35 (1.13–1.63)*^f^	1.85 (1.51–2.27)*^f^	--	1.34 (1.06–1.7)*	1.78 (1.43–2.23)*
*DMFT* count (RR)	Middle deprivation	1.23 (0.91–1.65), total *n*=543	1.27 (0.75–2.14), total *n*=3014	1.23 (0.95–1.58), total *n*=2544	--	0.78 (0.46–1.34), total *n*=2122	1.22 (0.78–1.91), total *n*=2431
	High deprivation	1.58 (0.93–2.7)	1.16 (0.76–1.77)	1.28 (1.03–1.59)*	--	0.83 (0.49–1.39)	1.44 (1.01–2.04)*
*DMFT* presence (OR)	Middle deprivation	1.22 (0.74–2.01), total *n*=543	1.18 (0.83–1.68), total *n*=3014	1.03 (0.78–1.34), total *n*=2544	--	1.99 (1.22–3.23)*^g^, total *n*=2122	1.03 (0.73–1.45)^g^, total *n*=2431
	High deprivation	1.53 (0.75–3.12)	1.2 (0.84–1.71)^h^	1.49 (1.14–1.95)*	--	2.46 (1.49–4.06)*^h^	1.43 (1.05–1.95)*
*Untreated decay* (OR)	Middle deprivation	1.14 (0.47–2.76), total *n*=543	1.32 (0.91–1.92), total *n*=3014	1.30 (0.99–1.71), total *n*=2544	--	1.44 (1.05–1.98)*, total *n*=2122	1.19 (0.83–1.71), total *n*=2431
	High deprivation	3.31 (1.16–9.45)*	2.03 (1.48–2.78)*	1.98 (1.47–2.68)*	--	1.65 (1.21–2.24)*	1.98 (1.48–2.63)*

*deft* = number of decayed, extracted (due to decay), and filled primary teeth; *DMFT* = number of decayed, missing (due to decay), and filled permanent teeth. *deft* or *DMFT* count = number of teeth with caries experience; *deft* or DMFT presence = presence (1 or more) versus absence of teeth with caries experience. *Untreated decay* = presence of two or more teeth (primary or permanent) with untreated decay

*RR*, rate ratio; *OR*, odds ratio

Separate regression models were run for each socioeconomic indicator–outcome variable combination

*Significantly different from the reference category at *p*<0.05, for that city

Within each row, the same lowercase superscript indicates statistically significant difference (*p*<0.05) between years (within cities) or between cities (within years) for the estimates indicated, as follows, with OR or RR (95% confidence interval) for comparison of coefficients:

^a^Association between no dental insurance and *deft* (count) in 2013 was lower in Calgary than in Edmonton, RR=0.81 (0.71–0.92)

^b^Association between no dental insurance and *DMFT* (count) in 2018 was lower in Calgary than in Edmonton, RR=0.67 (0.48–0.93)

^c^Association between no dental insurance and odds of *untreated decay* in Calgary was greater in 2018 than in 2009, OR=1.89 (1.36–2.63)

^d^Association between no dental insurance and odds of *untreated decay* in Calgary was greater in 2018 than in 2013, OR=1.67 (1.22–2.28)

^e^Association between no dental insurance and odds of *untreated decay* in 2018 was greater in Calgary than in Edmonton, OR=1.44 (1.03–2.02)

^f^Association between high (vs low) material deprivation and odds of *deft* in Calgary was greater in 2018 than in 2013, OR=1.37 (1.06–1.77)

^g^Association between middle (vs low) material deprivation and odds of *DMFT* in Edmonton was lower in 2018 than in 2013, OR=0.52 (0.29–0.92)

^h^Association between high (vs low) material deprivation and odds of *DMFT* in 2013 was lower in Calgary than in Edmonton, OR=0.49 (0.26–0.90)

Focusing first on associations in 2018/2019, there was statistical evidence of social inequities in dental caries in both cities at that most recent data point. For example, the odds of *untreated decay* were higher among those with no dental insurance than among those with dental insurance in both Calgary (odds ratio [OR]=3.34, 95% CI 2.64–4.22, *p*<0.05) and Edmonton (OR=2.31 [1.82–2.95], *p*<0.05) in 2018/2019. Relative to those in the lowest material deprivation tertile, those in the highest tertile (most deprivation) had higher levels/odds of all dental indicators (*deft*, *DMFT*, and *untreated decay*) in both Calgary and Edmonton. For example, the odds of *deft* (presence) among those in the highest, versus the lowest, material deprivation tertile were 1.85 (95% CI 1.51–2.27), *p*<0.05 in Calgary, and 1.78 (1.43-2.23), *p*<0.05 in Edmonton in 2018/2019.

There was statistical evidence that several associations persisted across city (Calgary and Edmonton) and over time (2009/2010, 2013/2014, and 2018/2019 for Calgary; 2013/2014 and 2018/2019 for Edmonton). These include the association between lack of dental insurance and higher odds of *untreated decay*, between higher material deprivation and higher *deft* (both count [RR] and presence [OR]), and between higher material deprivation and higher odds of *untreated decay*.

Certain differences between cities and waves were observed and are indicated in Table 2 using lowercase superscripts. To structure our presentation of results, we focus on differences between 2018/2019 (later post-cessation) and previous waves in both cities, and between Calgary (fluoridation cessation) and Edmonton (still fluoridated) in the 2018/2019 (later post-cessation) wave, because these differences (or lack thereof) are pertinent to our focus on presence/absence of fluoridation and oral health inequities. In Calgary (fluoridation cessation), the association between lack of dental insurance and higher odds of *untreated decay* in 2018/2019 (later post-cessation) was higher than the association in 2009/2010 (pre-cessation) (OR for comparison of coefficients = 1.89 [1.36–2.63], *p*<0.001) and it was higher than the association in 2013/2014 (early post-cessation) (OR for comparison of coefficients = 1.67 [1.22–2.28], *p*=0.001). Moreover, that same association (between lack of dental insurance and higher odds of *untreated decay*) was higher in Calgary (fluoridation cessation) in 2018/2019 than in Edmonton (still fluoridated) (OR for comparison of coefficients = 1.44 [1.03–2.02], *p*=0.033). The association in Calgary (fluoridation cessation) between higher material deprivation and higher odds of *deft* was higher in 2018/2019 (later post-cessation) than in 2013/2014 (early post-cessation) (OR for comparison of coefficients = 1.37 [1.06–1.77], *p*=0.016). In contrast, the association between lack of dental insurance and higher levels of *DMFT* (count) in 2018/2019 was lower in Calgary (fluoridation cessation) than in Edmonton (still fluoridated) (OR for comparison of coefficients = 0.67 [0.48–0.93], *p*=0.018).

Table 3 shows concentration indices in Calgary (2009/2010, 2013/2014, 2018/2019) and Edmonton (2013/2014 and 2018/2019 only). In 2018/2019, there was statistical evidence that all dental caries indicators were disproportionately concentrated among those with greater small area material deprivation in both Calgary (fluoridation cessation) and Edmonton (still fluoridated). Following Koolman and van Doorslaer (2004), we estimated (by multiplying the concentration index by 75) the percentage of each dental indicator that would need to be redistributed to arrive at an equal distribution. These estimates show considerable and similar departure from equality in both cities in 2018/2019: in Calgary, 6.4% (*deft*), 8.3% (*DMFT*), and 11.3% (*untreated decay*) would have to be redistributed from the higher to the lower half of the material deprivation distribution to achieve an equal distribution. In Edmonton, the values are 9% for *deft*, 9% for *DMFT*, and 9.8% for *untreated decay*. There were no differences (at *p*<0.05) in concentration indices between time points in each city (i.e., 2018/2019 vs 2013/2014 vs 2009/2010 in Calgary; 2018/2019 vs 2013/2014 in Edmonton) nor between cities at post-cessation (i.e., 2013/2014 vs 2018/2019).

**Table 3:** Concentration indices (with 95% confidence interval) showing the extent to which dental caries indicators (*deft*, *DMFT*, *untreated decay*) are disproportionately concentrated among those with greater small area material deprivation scores, Grade 2 schoolchildren in Calgary (2009/2010, 2013/2014, 2018/2019) and Edmonton (2013/2014 and 2018/2019)

**Outcome variable**	**Concentration index, 95% confidence interval,** ***n***					
	**Calgary** **(Fluoridation cessation in 2011)**			**Edmonton** **(Still fluoridated)**		
	**2009/2010**	**2013/2014**	**2018/2019**	**2009/2010**	**2013/2014**	**2018/2019**
*deft*	−0.065 (−0.13 to −0.003), *n*=511*	−0.082 (−0.11 to −0.06), *n*=2980*	−0.085 (−0.058 to −0.11), *n*=2564*	--	−0.085 (−0.054 to −0.12), *n*=2154*	−0.12 (−0.16 to −0.088), *n*=2459*
*DMFT*	−0.14 (−0.31 to 0.04), *n*=505	−0.031 (−0.12 to 0.055), *n*=2939	−0.11 (−0.032 to −0.18), *n*=2544*	--	−0.19 (−0.076 to −0.030), *n*=2112*	−0.12 (−0.19 to −0.039), *n*=2431*
*Untreated decay*	−0.055 (−0.09 to −0.019), *n*=505*	−0.036 (−0.48 to −0.24), *n*=2939*	−0.15 (−0.092 to −0.20), *n*=2500*	--	−0.083 (−0.033 to −0.13), *n*=2112	−0.13 (−0.19 to −0.080), *n*=2431*

*deft* = number of decayed, extracted (due to decay), and filled primary teeth; *DMFT* = number of decayed, missing (due to decay), and filled permanent teeth; *untreated decay* = presence of two or more teeth (primary or permanent) with untreated decay

*Index differs significantly from zero (equality) at *p*<0.05

There were no differences between cities (Calgary vs Edmonton) in 2013/2014 or 2018/2019, and there were no within-city differences over time (i.e., 2009/2010 vs 2013/2014 vs 2018/2019 for Calgary; 2013/2014 vs 2018/2019 for Edmonton)

Table 4 shows results of regression analyses in Calgary (fluoridation cessation) and Edmonton (still fluoridated) in 2013/2014 and 2018/2019 for the subset of socioeconomic variables that were only available at post-cessation (cessation occurred in Calgary in 2011). Several associations were consistent (statistically significant at *p*<0.05) across city (Calgary and Edmonton) and year (2013/2014 and 2018/2019). These include the association between public (vs private/employer) dental insurance and higher *deft* (both count [RR] and presence [OR]); between no insurance (vs private/employer insurance) and higher odds of *deft*; between no insurance or public insurance (vs private/employer insurance) and higher odds of *untreated decay*; between the lowest (vs the highest) level of household education and higher *deft* count (RR), between renting one’s home (vs owning with no mortgage) and higher *deft* (both count [RR] and presence [OR]), and between renting one’s home (vs owning with no mortgage) and higher odds of *untreated decay*.

**Table 4:** Weighted estimates from regression analyses (zero-inflated Poisson or logistic) among Grade 2 schoolchildren in Calgary and Edmonton using socioeconomic variables available at post-cessation only (2013/2014 and 2018/2019). Dental caries measures (*deft*, *DMFT*, *untreated decay*) regressed on categories of (1) dental insurance, (2) household education, and (3) dwelling tenure

**Outcome variable**	**Predictor variable**	**Estimate (RR or OR), 95% confidence interval,** ***n***			
		**Calgary** **(Fluoridation cessation in 2011)**		**Edmonton** **(Still fluoridated)**	
		**2013/2014**	**2018/2019**	**2013/2014**	**2018/2019**
*Dental insurance (reference: private/employer insurance)*					
*deft* count (RR)	No insurance	0.99 (0.9–1.08)^a^, total *n*=3164	1.08 (0.99–1.18), total *n*=2569	1.21 (1.1–1.32)*^a^, total *n*=2196	1.1 (0.99–1.22), total *n*=2514
	Public insurance	1.32 (1.21–1.44)*	1.17 (1.08–1.28)*	1.22 (1.11–1.36)*	1.13 (1.01–1.25)*
*deft* presence (OR)	No insurance	1.24 (1.0–1.52)*, total *n*=3164	1.39 (1.07–1.8)*, total *n*=2569	1.47 (1.14–1.89)*, total *n*=2196	1.49 (1.14–1.93)*, total *n*=2514
	Public insurance	2.31 (1.74–3.07)*	2.09 (1.43–3.05)*	1.88 (1.46–2.44)*^b^	3.08 (2.18–4.37)*^b^
*DMFT* count (RR)	No insurance	1.56 (1.03–2.37), total *n*=3120*	0.91 (0.68–1.22)^c^, total *n*=2548	1.5 (0.96–2.33), total *n*=2153	1.38 (1.01–1.89)*^c^, total *n*=2486
	Public insurance	1.01 (0.61–1.69)	1.16 (0.86–1.57)	1.38 (0.89–2.14)^d^	0.93 (0.65–1.33)^d^
*DMFT* presence (OR)	No insurance	1.24 (0.82–1.87), total *n*=3120	1.17 (0.84–1.64), total *n*=2548	1.7 (1.07–2.7)*, total *n*=2153	1.36 (0.94–1.96), total *n*=2486
	Public insurance	1.64 (1.08–2.49)*	1.61 (1.04–2.5)*	2.37 (1.52–3.69)*	1.39 (0.96–2.02)
*Untreated decay* (OR)	No insurance	2.18 (1.71–2.79)*^e^, total *n*=3120	3.75 (2.9–4.85)*^e,f^, total *n*=2548	2.23 (1.67–2.96)*, total *n*=2153	2.53 (1.98–3.23)*^f^, total *n*=2486
	Public insurance	1.87 (1.29–2.71)*	2.15 (1.53–3.04)*	1.6 (1.14–2.25)*	1.85 (1.36–2.51)*
*Household education (reference: completed degree or certificate higher than bachelor’s [highest category])*					
*deft* count (RR)	Low	1.15 (1.04–1.26)*, total *n*=3092	1.23 (1.13–1.33)*, total *n*=2562	1.12 (1.02–1.24)*, total *n*=2145	1.12 (1.0–1.24)*, total *n*=2529
	Middle	1.13 (1.0–1.28)	1.08 (1.0–1.18)	1.05 (0.93–1.19)	1.09 (0.97–1.22)
*deft* presence (OR)	Low	1.46 (1.22–1.76)*^g^, total *n*=3092	2.19 (1.78–2.7)*^g,h^, total *n*=2562	1.21 (0.95–1.54), total *n*=2145	1.46 (1.17–1.81)*^h^, total *n*=2529
	Middle	1.27 (1.03–1.56)*	1.39 (1.09–1.78)*	1.03 (0.8–1.34)	1.18 (0.94–1.46)
*DMFT* count (RR)	Low	1.02 (0.67–1.57), total *n*=3049	1.3 (0.98–1.71), total *n*=2541	1.26 (0.68–2.32), total *n*=2103	0.91 (0.62–1.35), total *n*=2501
	Middle	0.98 (0.58–1.66)	1.25 (0.92–1.69)	1.24 (0.64–2.44)	1.01 (0.67–1.54)
*DMFT* presence (OR)	Low	1.4 (1.01–1.95)*, total *n*=3049	1.66 (1.22–2.25)*, total *n*=2541	1.24 (0.82–1.88), total *n*=2103	1.1 (0.78–1.56), total *n*=2501
	Middle	1.21 (0.8–1.83)	1.19 (0.92–1.54)	0.65 (0.37–1.16)	0.94 (0.66–1.35)
*Untreated decay* (OR)	Low	1.64 (1.2–2.23)*, total *n*=3049	2.13 (1.58–2.87)*^i^, total *n*=2541	1.34 (0.98–1.84), total *n*=2103	1.24 (0.94–1.63)^i^, total *n*=2501
	Middle	1.27 (0.9–1.79)	1.44 (1.05–1.98)*	1.15 (0.83–1.6)	1.03 (0.77–1.36)
*Dwelling tenure (reference: owned, no mortgage)*					
*deft* count (RR)	Rent or other	1.21 (1.06–1.38)*, total *n*=3090	1.2 (1.05–1.37)*, total *n*=2534	1.25 (1.05–1.49)*, total *n*=2135	1.31 (1.06–1.62)*, total *n*=2466
	Own with mortgage	1.09 (0.96–1.25)	1.1 (0.97–1.24)	1.02 (0.84–1.22)	1.09 (0.88–1.35)
*deft* presence (OR)	Rent or other	1.92 (1.49–2.49)*, total *n*=3090	1.6 (1.08–2.37)*, total *n*=2534	1.73 (1.19–2.51)*, total *n*=2135	2.43 (1.69–3.49)*, total *n*=2466
	Own with mortgage	1.32 (1.04–1.67)*	1.16 (0.81–1.66)	1.04 (0.73–1.48)	1.34 (0.97–1.85)
*DMFT* count (RR)	Rent or other	1.55 (0.8–3.0), total *n*=3046	1.19 (0.63–2.26), total *n*=2513	1.43 (0.68–3.03), total *n*=2094	1.14 (0.73–1.78), total *n*=2440
	Own with mortgage	1.19 (0.61–2.35)	1.1 (0.55–2.22)	0.99 (0.43–2.29)	0.87 (0.56–1.36)
*DMFT* presence (OR)	Rent or other	1.74 (1.03–2.94)*, total *n*=3046	2.11 (1.3–3.43)*, total *n*=2513	1.96 (1.2–3.2)*, total *n*=2094	1.4 (0.83–2.38), total *n*=2440
	Own with mortgage	1.01 (0.6–1.7)	1.68 (1.03–2.75)*	0.97 (0.55–1.71)	1.06 (0.63–1.79)
*Untreated decay* (OR)	Rent or other	3.29 (2.05–5.27)*, total *n*=3046	2.68 (1.69–4.24)*, total *n*=2513	2.35 (1.5–3.68)*, total *n*=2094	2.21 (1.33–3.68)*, total *n*=2440
	Own with mortgage	1.66 (1.1–2.5)*	1.15 (0.72–1.86)	1.11 (0.73–1.69)	1.26 (0.77–2.06)

*deft* = number of decayed, extracted (due to decay), and filled primary teeth; *DMFT* = number of decayed, missing (due to decay), and filled permanent teeth. *deft* or *DMFT* count = number of teeth with caries experience; *deft* or DMFT presence = presence (1 or more) versus absence of teeth with caries experience. *Untreated decay* = presence of two or more teeth (primary or permanent) with untreated decay

Household education categories: low: ≤ high school grad or post-secondary diploma or certificate; middle: completed bachelor’s degree; high: completed degree or certificate higher than bachelor’s

*RR*, rate ratio; *OR*, odds ratio; *95% CI*, 95% confidence interval

Separate regression models were run for each socioeconomic indicator–outcome variable combination

*Effect is statistically significant (relative to reference category) at *p*<0.05

Within each row, the same lowercase superscript indicates statistically significant difference (*p*<0.05) between years (within cities) or between cities (within years) for the estimates indicated, as follows, with OR or RR (95% confidence interval) for comparison of coefficients:

^a^Association between no dental insurance (vs private/employer insurance) and *deft* (count) was lower in Calgary than in Edmonton, RR=0.82 (0.72–0.93)

^b^Association between public dental insurance (vs private/employer insurance) and odds of *deft* in Edmonton was higher in 2018 than in 2013, OR=1.64 (1.05–2.55)

^c^Association between no dental insurance (vs private/employer insurance) and *DMFT* (count) in 2018 was lower in Calgary than in Edmonton, RR=0.69 (0.49–0.98)

^d^Association between public dental insurance (vs private/employer insurance) and *DMFT* (count) in Edmonton was lower in 2018 than in 2013, RR=0.54 (0.32–0.89)

^e^Association between no dental insurance (vs private/employer insurance) and odds of *untreated decay* in Calgary was greater in 2018 than in 2013, OR=1.72 (1.24–2.39)

^f^Association between no dental insurance (vs private/employer insurance) and odds of *untreated decay* in 2018 was greater in Calgary than in Edmonton, OR=1.48 (1.04–2.12)

^g^Association between low (vs high) household education and odds of *deft* in Calgary was greater in 2018 than in 2013, OR=1.50 (1.14–1.97)

^h^Association between low (vs high) household education and odds of *deft* in 2018 was greater in Calgary than in Edmonton, OR=1.51 (1.11–2.03)

^i^Association between low (vs high) household education and odds of *untreated decay* in 2018 was greater in Calgary than in Edmonton, OR=1.72 (1.15–2.58)

Differences between cities at each time point, and between time points in each city are denoted using lower-case letter superscripts in Table  4. To structure our presentation of results, we focus on differences between 2018/2019 (later post-cessation) and 2013/2014 (early post-cessation), and between Calgary (fluoridation cessation) and Edmonton (still fluoridated) in the 2018/2019 (later post-cessation) wave, because these differences (or lack thereof) are pertinent to our focus on presence/absence of fluoridation and oral health inequities. In 2018/2019, there was statistical evidence that the association between no dental insurance (relative to private/employer insurance) and higher odds of *untreated decay* and the association between low (vs high) household education and higher odds of *deft* and of *untreated decay* were higher in Calgary (fluoridation cessation) than in Edmonton (still fluoridated) (OR for comparison of coefficients = 1.48 [95% CI 1.04–2.12], *p*=0.029 for the no dental insurance*–untreated decay* association; 1.51 [1.11–2.03], *p*=0.008 for the low education*–deft* (presence) association; and 1.72 [1.15–2.58], *p*=0.009 for the low education*–untreated decay* association. Moreover, in Calgary (fluoridation cessation), the association between no dental insurance (vs private/employer insurance) and higher odds of *untreated decay* and the association between low (vs high) household education and higher odds of *deft* were greater in 2018/2019 (later post-cessation) than in 2013/2014 (early post-cessation) (OR for comparison of coefficients = 1.72 [1.24–2.39], *p*=0.001 for the no dental insurance*–untreated decay* association; and 1.50 [1.14–1.97] *p*=0.004 for the low education*–deft* [presence] association). In contrast, the association between public (vs private/employer) dental insurance and higher odds of *deft* in Edmonton (still fluoridated) was greater in 2018/2019 than in 2013/2014 (OR for comparison of coefficients = 1.64 [1.05–2.55], *p*=0.03); and the association between no dental insurance (vs private/employer insurance) and *DMFT* (count) in 2018/2019 was lower in Calgary (fluoridation cessation) than in Edmonton (still fluoridated) (RR for comparison of coefficients = 0.69 [0.49–0.98], *p*=0.039).

## Discussion

We examined social inequities in children’s dental caries (tooth decay) in the cities of Calgary and Edmonton, Canada, in the context of fluoridation cessation which occurred in Calgary in 2011.

Main findings are twofold. First, our findings reveal persistent social inequities in dental caries in our setting. For both Calgary and Edmonton samples in the post-cessation period (2013/2014, 2018/2019), several socioeconomic indicators (i.e., no or public dental insurance, greater small area material deprivation, lower household educational attainment, and renting vs owning one’s home) were consistently associated with poorer dental health, including dental caries experience in primary teeth (*deft*) and untreated decay. Our findings thus corroborate the considerable amount of evidence showing social inequities in children’s dental health (Schwendicke et al., 2015), which is important because these inequities are both unfair and avoidable. There were fewer and less consistent findings for dental caries experience in permanent teeth (*DMFT*). This likely reflects the age of children in our study (approx. age 7) and the limited amount of time that their permanent teeth had been exposed to the oral environment (McLaren et al., 2021; Kuthy & Ashton, 1989).

Second, we observed some differences between cities and survey waves, which shed light on the effect of fluoridation cessation on children’s dental health equity. It is important to note that some observed differences were not consistent with an adverse effect of fluoridation cessation. Specifically, the association between no dental insurance and higher caries experience in permanent teeth (*DMFT* count) in 2018/2019 was greater in Edmonton (still fluoridated) than in Calgary (fluoridation cessation); and the association between public (vs private/employer) dental insurance and higher odds of caries experience in primary teeth (*deft* presence) in Edmonton was greater in 2018/2019 than in 2013/2014 (indicating an increasing inequity over time in a setting where fluoridation was consistently present).

Far more often, however, observed differences between cities and survey waves were consistent with an adverse effect of fluoridation cessation on dental health inequities. Specifically, several associations in Calgary (fluoridation cessation), using different socioeconomic indicators, increased over time, indicating increasing inequities with increasing time since fluoridation cessation. These included the association between no dental insurance and higher odds of *untreated decay*, between higher material deprivation and higher odds of dental caries in primary teeth (*deft* presence), and between low household education and higher odds of dental caries in primary teeth (*deft* presence). Moreover, several associations, again using different socioeconomic indicators, in 2018/2019 (later post-cessation) were greater in Calgary (fluoridation cessation) than in Edmonton (still fluoridated). These included the association between no dental insurance and higher odds of *untreated decay*, and between low household education and both higher odds of dental caries in primary teeth (*deft* presence) and higher odds of *untreated decay*. Our interpretation (that these differences are consistent with an adverse effect of fluoridation cessation) is further supported by the fact that all of these associations pertain to dental caries in primary teeth—which, as noted above, our study was better designed to detect (compared to dental caries in permanent teeth)—and to untreated decay, which is consistent with fluoridation as a primary prevention activity—that is, an intervention that influences incidence of disease.

One important study weakness is the limitations of the single pre-cessation (2009/2010) data point, which was only available for Calgary. A 2015 systematic review (Iheozor-Ejiofor et al., 2015) concluded that there was insufficient information to determine whether fluoridation reduces differences in tooth decay between children from different socioeconomic backgrounds. Importantly, our study would not qualify for inclusion in the Cochrane review because of this limitation (the review stipulated inclusion of a comparison group at both pre- and post-cessation periods). Our single pre-cessation data point was moreover limited by a smaller sample and limited socioeconomic information, which is in line with its collection for surveillance purposes rather than research. Another limitation is that, because we tested associations for each socioeconomic indicator separately, we cannot comment on whether or how socioeconomic indicators may interrelate (e.g., interact) to influence dental caries, which may be important for understanding social inequities in dental health, including implications for policy and practice. Finally, and importantly, this study did not directly consider numerous other factors that may contribute to social inequities in dental health, including those which may differ between our two cities (although see McLaren et al., 2021). It is important for future research on this topic to embrace a multifaceted approach which considers social determinants of oral health inequities operating at various levels from the individual to the global (Watt, 2012).

Our study nonetheless contributes to existing literature on fluoridation and social inequities in dental caries because it includes comparisons over multiple time points (vs a single cross-sectional survey) and it sheds light on the practical question of whether or the extent to which other interventions (e.g., other dental public health programs) in our setting have been adequate to offset the lack of fluoridation in Calgary. Other strengths include the population-representative samples, the high-quality oral health data collected by trained and calibrated experts, and multiple indicators of socioeconomic circumstances, especially at post-cessation, which permits some assessment of consistency and specificity of effects.

## Conclusion

We observed significant and persistent social inequities in dental health among children in both Calgary (fluoridation cessation) and Edmonton (still fluoridated), thus providing a reminder of the importance of building equity considerations into public health policy and practice. When we observed differences between cities or between survey waves, they were usually in the direction of being worse in Calgary (where fluoridation was ceased) than in Edmonton, suggesting that fluoridation cessation may have contributed to worsening inequities in dental health in our setting. Our findings are consistent with existing cross-sectional studies showing an association between fluoridation and greater dental health equity, using the relatively under-exploited research opportunity presented by a policy decision to cease fluoridation (McLaren & Singhal, 2016). Within the context of a multifaceted approach to dental public health, decisions about fluoridation should include consideration of its health equity impact, alongside evidence on population-wide benefits, safety, and other dimensions (Nuffield Council on Bioethics, 2007). Our findings may be particularly relevant to other settings where, similar to our setting of Alberta, Canada, income inequality is high (Flanagan, 2015), dental services (which are overwhelmingly financed and delivered in the private sector) are costly (Quiñonez, 2020), and dental public health infrastructure is limited and programs are targeted in nature (Alberta Health Services, 2016; Huber et al., 2017; McLaren & Petit, 2018).

## Contributions to knowledge

What does this study add to existing knowledge?

- Social inequities in children’s dental health are significant and avoidable. Community water fluoridation, because of its universal scope and passive mechanism of uptake, is one element of a multifaceted approach to promoting dental health equity.
- We studied children’s dental health inequities in the context of fluoridation cessation, which presents an under-used research opportunity. We observed social inequities in children’s dental caries in both Calgary (fluoridation cessation) and Edmonton (still fluoridated), Canada. However, some post-cessation associations were larger in Calgary than in Edmonton, and increased in Calgary over time since cessation, suggesting that fluoridation cessation may have led to a worsening in dental health equity.

What are the key implications for public health interventions, practice or policy?

- Our results suggest that social inequities in children’s dental health are significant, and that discontinuing fluoridation may contribute to widening inequities in children’s dental health.
- Dental health equity should be a key concern for communities that are thinking about discontinuing fluoridation. This is especially true in settings like ours where income inequality is high, dental services are costly, and dental public health infrastructure is limited.

## Footnotes

1 These sample sizes are for dental caries experience in primary teeth (i.e., *deft*). Sample sizes are lower, as seen in the tables below, for permanent teeth (due to insufficient eruption in some children) and for analysis of social inequities (due to partial missing data).

2 Two or more decayed teeth was a pragmatic choice intended to achieve a balance between seriousness of the problem and adequate number of children (the proportion with 3 or more decayed teeth was considerably smaller).

3 Government programs in our setting at the time of the study were targeted in nature, with eligibility based on low-income or Indigenous status. Provincial programs include the Alberta Child Health Benefit, Supports for Independence, AISH (and dependants), Guardianship Orders, and Alberta Works; the main federal program is Non-Insured Health Benefits for registered First Nations and recognized Inuit persons.

4 In addition to producing rates, the Zero-Inflated Poisson (ZIP) regression produces results from the excess zeros (logistic regression) component of the model, in the form of odds ratios (OR) for absence of *deft/DMFT*. To simplify interpretation, we analyzed these components using separate logistic regression for presence (1 or more) versus absence (zero) of *deft* and *DMFT* on the various socioeconomic indicators.

5 We transformed the variable so that a negative concentration, as per convention, corresponds to greater concentration of dental caries among lower socioeconomic groups (in our case, higher material deprivation).
